# Lead-Poisoning among Compositors

**Published:** 1898-09-10

**Authors:** 


### LEAD-POISONING AMONG COMPOSITORS.

The reports of the inspectors of factories in Austria and Germany show that in the composing-rooms of printing-houses the dust is often charged with lead to such an extent that it becomes a matter of some surprise that lead-poisoning is not more common among compositors than seems to be the case. The dust from the floor was frequently found to contain as much as 10 to 12 per cent. by weight of lead, while that collected from the compartments of the "case" itself contained no less than 16·5 per cent. The weight of the particles, however, causes them to fall quickly to the ground, so that there is but rarely more than about 1 per cent. of lead in dust collected from a few feet above it, and it seems probable that a careful removal of the dust from the floors by mops or damp cloths, together with a little care in keeping the "case" free from accumulations of dust, would be sufficient to remove most of the risk of lead-poisoning, except such as may arise from direct contact with the type and from eating without previously washing the hands.

